# Structurally Related Monoterpenes *p*-Cymene, Carvacrol and Thymol Isolated from Essential Oil from Leaves of *Lippia sidoides* Cham. (Verbenaceae) Protect Mice against Elastase-Induced Emphysema

**DOI:** 10.3390/molecules21101390

**Published:** 2016-10-20

**Authors:** Ellen Games, Marina Guerreiro, Fernanda R. Santana, Nathalia M. Pinheiro, Emerson A. de Oliveira, Fernanda D.T.Q.S. Lopes, Clarice R. Olivo, Iolanda F.L.C. Tibério, Mílton A. Martins, João Henrique G. Lago, Carla M. Prado

**Affiliations:** 1 Department of Biological Science, Universidade Federal de São Paulo, Diadema 09913-030, Brazil; ellengames@gmail.com (E.G.); marinaguerreiro88@gmail.com (M.G.); fe.paula.roncon@gmail.com (F.R.S.); dupontemerson@hotmail.com (E.A.d.O.); 2 Department of Medicine, School of Medicine, Universidade de São Paulo, São Paulo 01246903, SP, Brazil; pinheiro.nathalia@gmail.com (N.M.P.); fernandadtqsl@gmail.com (F.D.T.Q.S.L.); clariceolivo@gmail.com (C.R.O.); iocalvo@uol.com.br (I.F.L.C.T.); mmartins@usp.br (M.A.M.); 3 Department of Exact Science and Earth, Universidade Federal de São Paulo, Diadema 09913-030, Brazil; joaohglago@gmail.com; 4 Center of Natural Sciences and Humanities, Federal University of ABC, Santo Andre 09606-045, SP, Brazil; 5 Department of Bioscience, Federal University of São Paulo, Campus Baixada Santista, Santos 11015-020, SP, Brazil

**Keywords:** COPD, elastase-induced emphysema, monoterpenes, *p*-cymene, carvacrol, thymol, lung inflammation

## Abstract

Background: Chronic obstructive pulmonary disease (COPD) is characterized by irreversible airflow obstruction and inflammation. Natural products, such as monoterpenes, displayed anti-inflammatory and anti-oxidant activities and can be used as a source of new compounds to COPD treatment. Our aim was to evaluate, in an elastase-induced pulmonary emphysema in mice, the effects of and underlying mechanisms of three related natural monoterpenes (*p*-cymene, carvacrol and thymol) isolated from essential oil from leaves *Lippia sidoides* Cham. (Verbenaceae). Methods: Mices received porcine pancreatic elastase (PPE) and were treated with *p*-cymene, carvacrol, thymol or vehicle 30 min later and again on 7th, 14th and 28th days. Lung inflammatory profile and histological sections were evaluated. Results: In the elastase-instilled animals, the tested monoterpenes reduced alveolar enlargement, macrophages and the levels of IL-1β, IL-6, IL-8 and IL-17 in bronchoalveolar lavage fluid (BALF), and collagen fibers, MMP-9 and p-65-NF-κB-positive cells in lung parenchyma (*p* < 0.05). All treatments attenuated levels of 8-iso-PGF2α but only thymol was able to reduced exhaled nitric oxide (*p* < 0.05). Conclusion: Monoterpenes *p*-cymene, carvacrol and thymol reduced lung emphysema and inflammation in mice. No significant differences among the three monoterpenes treatments were found, suggesting that the presence of hydroxyl group in the molecular structure of thymol and carvacrol do not play a central role in the anti-inflammatory effects.

## 1. Introduction

Pulmonary emphysema is a main constituent of chronic obstructive pulmonary disease (COPD), which is associated to high morbidity and mortality in worldwide but commonly preventable and treatable. It is characterized by alveolar destruction and abnormal permanent enlargement of the alveoli which leads to severe airflow limitation [1]. Inflammatory cells such as neutrophils, macrophages and lymphocytes are activated and release several pro-inflammatory mediators such as interleukin IL1-β, IL-6 and IL-8 [2,3,4,5,6]. Recently, new interleukins had their role recognized which are involved in a wide variety of inflammatory response such as IL-15, IL-18, IL-21, IL-22, IL-23, IL-27, and IL-33 [7]. Commonly, IL-17, which has been become attention since it is produced in a particularly subset of T Helper cells called Th17, is involved in sustaining the inflammation amplifying the inflammatory responses [7]. Moreover, the cyclo-oxygenases that control the production of prostaglandins and thromboxanes [8] are also involved in the modulation of lung inflammation and a flavone chrysin suppressed inflammatory response by the interaction with COX-2 binding site [9]. In addition, the translocation of nuclear transcription factor NF-κB to the nucleus up-regulates the transcription of several cytokines and pro-inflammatory mediators [10,11,12]. The main cause of COPD is the inhalation of cigarette smoke [13] which causes an increase in oxidative stress that plays a central role in this disease because it is both generated due to increased reactive oxygen and nitrogen species induced by smoke as well as produced by activated inflammatory cells [13,14]. Isoprostanes are generated at the end of the membrane lipid peroxidation cascade and has been used as an indirect marker of oxidative stress [15,16,17,18]. Moreover, exhaled nitric oxide, which is mainly produced by inducible nitric oxide enzyme (iNOS) in inflammatory situations, could also have been used as an inflammatory and oxidative stress marker, particularly if it is associated to other parameters [19,20]. Other mechanism involved in emphysema is the imbalance between protease and antiprotease that is responsible for the lung destruction. Neutrophil elastase, metalloproteases and cathepsins are responsible for the degradation of the proteins in alveolar wall extracellular matrix, such as collagen and elastin fibers, thereby causing the lung destruction. Oxidative stress is also involved in the inhibition of the antiprotease activities. The destruction and repair process in response to the presence of chronic inflammation and oxidative stress culminate in a remodeling process which is characterized by an increase in collagen deposition and broken of elastic fibers compromising the mechanical properties of the lung [21,22].

Medicinal plants and their constituents have been widely used to treat several diseases since ancient times and many natural products have been related to effectively counteract lung inflammation in acute and chronic models of lung disease [23,24]. In this regard, drugs that present anti-inflammatory properties have been extensively studied since unwanted side effects are associated to the traditional treatments for inflammation [23,24]. *p*-Cymene, carvacrol and thymol are structurally related aromatic monoterpenes obtained from essential oils from different plant species [25]. Thymol and carvacrol are phenolic compounds belonging to a class of natural antioxidants due to the presence of one hydroxyl group linked to aromatic ring and the possibility of stabilizing free radicals formation [26], which is associated to preventing lipid peroxidation [27]. A related monoterpene derivative, *p*-cymene, displayed a similar structure of thymol and carvacrol, but the hydroxyl group is absent in the aromatic ring. Considering that these compounds displayed different biological activities, including antitumoral, antibacterial, antifungal, anti-inflammatory, and antioxidant activities [28,29,30], the aim of the present study was to investigate the effects and underlying mechanisms of natural monoterpenes *p*-cymene, carvacrol and thymol, isolated from essential oil from leaves *Lippia sidoides* (Verbenaceae) in an elastase-induced pulmonary emphysema model in mice.

## 2. Results

### 2.1. Chemical Analysis of Isolated Monoterpenes from Essential Oil from Leaves of L. sidoides

**Figure 1:**
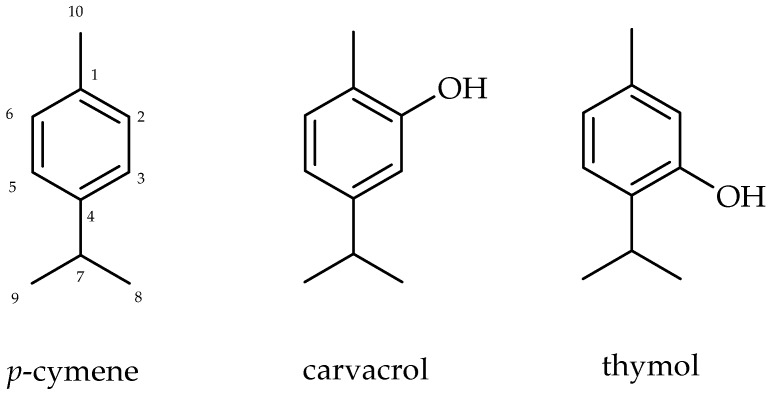
Structure of monoterpenes *p*-cymene, carvacrol and thymol.

The ^13^C-NMR spectra of three isolated monoterpenes from essential oil from leaves of *L. sidoides* showed peaks of sp^2^ carbons at range δ 112–154 assigned to aromatic ring (C-1 to C-6) and methyl group linked to aromatic ring at δ 15–21 (C-10). Additionally, the presence of two equivalent methyl groups at δ 22–24 and one methyne carbon at δ 26–37 indicated the presence of one isopropyl group. The comparison of obtained spectral data with those reported in the literature [31], associated to LREIMS analysis [32], allowed the identification of the monoterpenes *p*-cymene, carvacrol and thymol, as shown in Figure 1.

### 2.2. Monoterpenes Reduced BALF Lung Inflammation in PPE-Induced Emphysema in Mice

**Figure 2:**
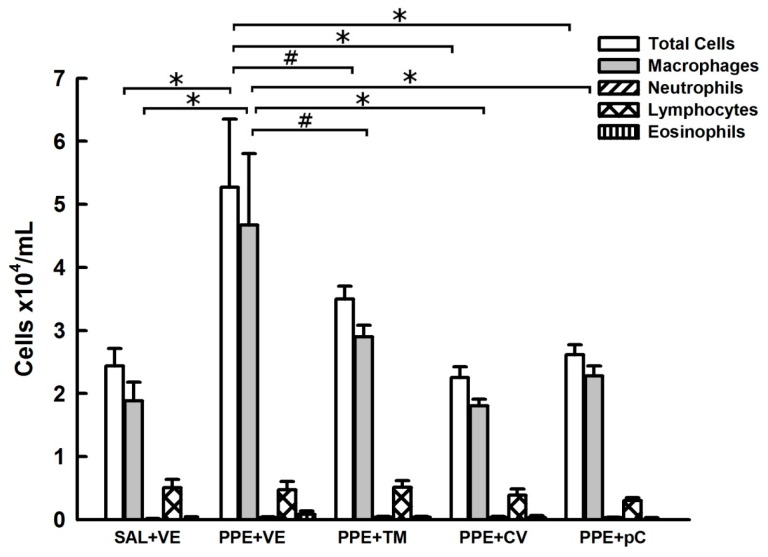
Effects of monoterpenes *p*-cymene, carvacrol and thymol on lung inflammation. Data are presented as mean ± standard error (SE) (*n* = 7 to 8 mice/group) of number of cells recovered in bronchoalveolar lavage fluid collected at the 28th day of the experimental protocol. SAL + VE: control group with vehicle treatment; PPE + VE: porcine pancreatic elastase instillation and vehicle treatment; PPE + TM: porcine pancreatic elastase instillation and thymol treatment; PPE + CV: porcine pancreatic elastase instillation and carvacrol treatment; PPE + pC: porcine pancreatic elastase instillation and *p*-cymene treatment. * *p* < 0.01 and ^#^ *p* < 0.05.

Figure 2 shows the number of total cells as well as the number of macrophages, neutrophils, lymphocytes, and eosinophils recovered in BALF. The PPE instillation increased inflammatory cells in BALF, particularly macrophages, compared to SAL + VE group (*p* < 0.01 for both comparisons). The treatment with monoterpenes *p*-cymene, carvacrol and thymol reduced the number of total cells and macrophages (PPE + CA and PPE + pC, *p* < 0.01 and PPE + TM, *p* < 0.05) in BALF compared to values obtained in PPE + VE group. No differences were detected in the number of neutrophils, lymphocytes and eosinophils among the experimental groups. 

### 2.3. Monoterpenes Reduced Cytokines in BALF in PPE-Induced Emphysema in Mice

**Figure 3:**
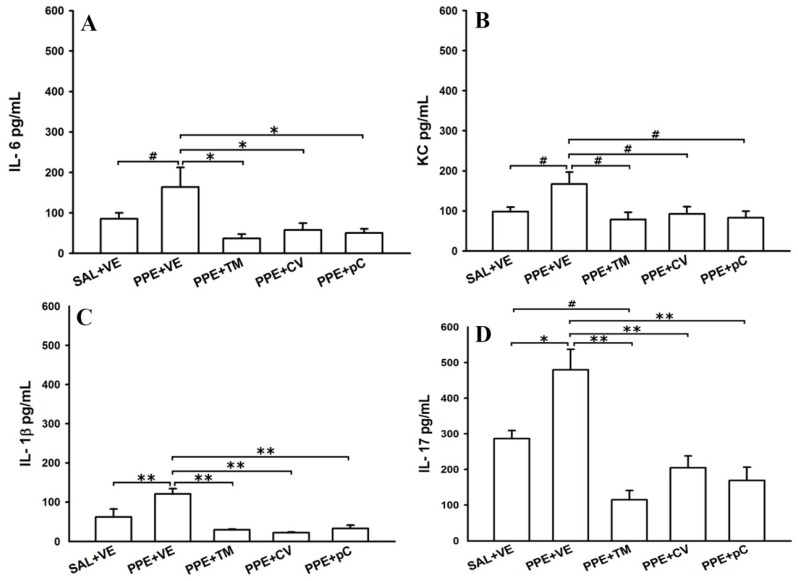
Effects of monoterpenes thymol, carvacrol and *p*-cymene in BALF cytokine levels. Data are presented as mean ± standard error (SE) (*n* = 6 to 8 mice/group) of levels of: IL-6 (**A**); KC (**B**); IL-1β (**C**); and IL-17 (**D**) detected in BALF of mice on the 28th day of the experimental protocol. SAL + VE: control group with vehicle treatment; PPE + VE: porcine pancreatic elastase instillation and vehicle treatment; PPE + TM: porcine pancreatic elastase instillation and thymol treatment; PPE + CV: porcine pancreatic elastase instillation and carvacrol treatment; PPE + pC: porcine pancreatic elastase instillation and *p*-cymene treatment. * *p* < 0.01, ** *p* < 0.001 and ^#^ *p* < 0.05.

To investigate the role of tested monoterpenes in modulating the secretion of cytokines in BALF, the levels of IL-6 (Figure 3A); KC, which is homologous to IL-8 in humans (Figure 3B); IL-1β (Figure 3C); and IL-17 (Figure 3D) were measured using ELISA. The PPE + VE group showed higher levels of IL-6 (*p* < 0.05), KC (*p* < 0.05), IL-1β (*p* < 0.001) and IL-17 (*p* < 0.01) compared to SAL + VE group. Moreover, the treatment with monoterpenes *p*-cymene, carvacrol and thymol reduced the levels of these cytokines in a similar mode to those observed in SAL + VE group (IL-6: *p* < 0.01; KC: *p* < 0.05; IL-1β and IL-17: *p* < 0.001 for the three treatments). Notwithstanding, the levels of IL-17 in thymol treated animals were also reduced in comparison to control animals (*p* < 0.05).

### 2.4. Monoterpenes Prevented Alveolar Septa Destruction Reduced the Collagen Fibers Deposition Probably by Controlling MMP-9/TIMP-1 in PPE-Induced Emphysema Model

**Figure 4:**
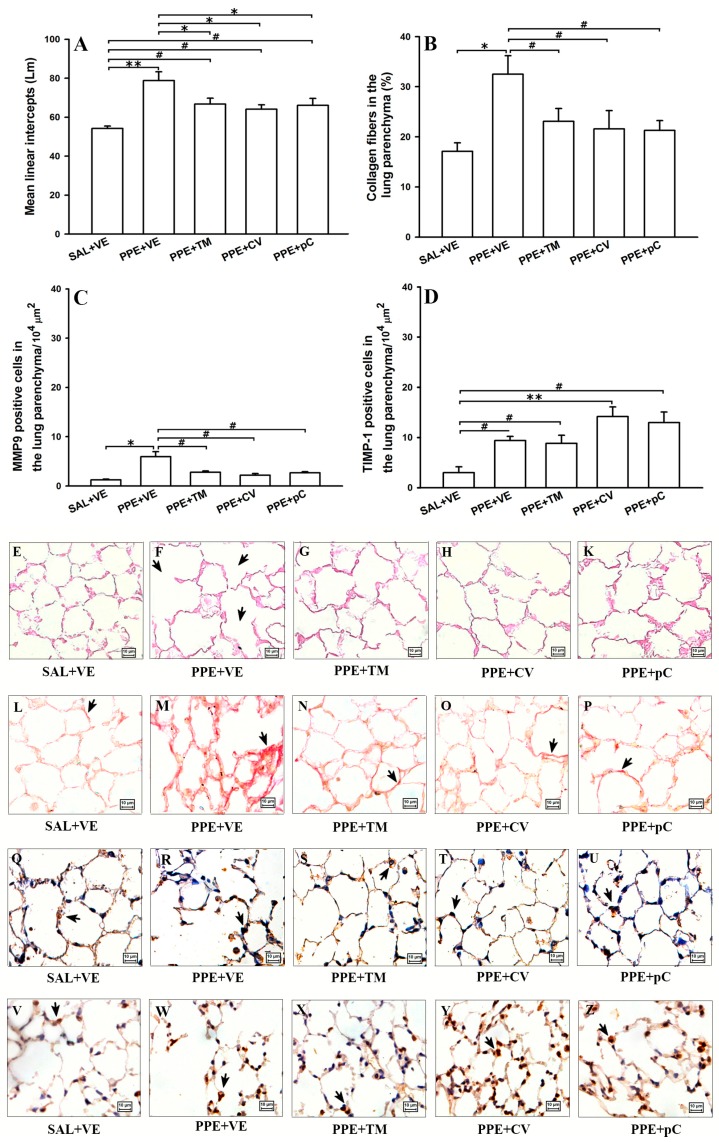
Effects of monoterpenes thymol, carvacrol and *p*-cymene on alveolar septa. Data are presented as mean ± standard error (SE) (*n* = 5 mice/group) of: mean linear intercepts (Lm) (**A**); volume proportion of collagen fibers (**B**); and the number of positive cells to MMP-9 (**C**); and TIMP-1 (**D**) in lung of mice in the 28th day of the protocol. The photomicrographs represent the lung tissue of one animal from control (**E**,**L**,**Q**,**V**), PPE treated with vehicle (**F**,**M**,**R**,**W**) and PPE treated with thymol (**G**,**N**,**S**,**X**), carvacrol (**H**,**O**,**T**,**Y**) or *p*-cymene group (**K**,**P**,**U**,**Z**) stained with HE (first line), picro-sirius to detect collagen fibers (second line), by immunohistochemistry to MMP-9 and TIMP-1 (third and fourth line respectively). Arrows indicate lung emphysema, collagen deposition and positive cells. SAL + VE: control group with vehicle treatment; PPE + VE: porcine pancreatic elastase instillation and vehicle treatment; PPE + TM: porcine pancreatic elastase instillation and thymol treatment; PPE + CV: porcine pancreatic elastase instillation and carvacrol treatment; PPE + pC: porcine pancreatic elastase instillation and *p*-cymene treatment. * *p* < 0.01, ** *p* < 0.001 and ^#^ *p* < 0.05.

The evaluation of lung emphysema and collagen deposition as well as the MMP-9/TIMP-1 balance is shown in Figure 4. The mean linear intercept (Lm) was used as indicator of alveolar destruction in mice. Mean Linear Intercepts (Lm) increased in PPE + VE group compared to the animals that received saline and vehicle treatment (SAL + VE) (*p* < 0.001) (Figure 4A). The tested monoterpenes attenuated the emphysema, as shown by the lower values of LM observed in PPE + TM, PPE + CV and PPE + pC groups (*p* < 0.01) compared with ELA + VE group. However, the treated animals showed high values of LM when compared to SAL + VE group (*p* < 0.05).

Due to degradation of the fibers to be usually followed by a turnover of collagen, collagen content (Figure 4B) in alveolar septa of mice was measured. The obtained results indicated an increase of proportion of collagen fibers in alveolar septa of ELA + VE animals compared with the SAL + VE group (*p* < 0.01) and the treatments reduced this response (*p* < 0.05). Since balance between MMPs and TIMPs is involved in the control of the degradation and turnover of the fibers, was quantified the expression of MMP-9 (Figure 4C) and TIMP-1 (Figure 4D) in cells presented in alveolar septa. The obtained results showed an increase of MMP-9 (*p* < 0.01) and TIMP-1 (*p* < 0.05) positive cells in alveolar septa from ELA + VE group compared with the control group (SAL + VE). Interestingly, the positive cells to MMP-9 were reduced in lung of the animals that received elastase and were treated with *p*-cymene, carvacrol and thymol (*p* < 0.05) compared with ELA + VE group. However, the number of positive cells to TIMP-1 in alveolar septa of animals that received elastase and treated with tested monoterpenes was similar to those observed in ELA + VE group.

The photomicrographs represent the lung tissue of one animal from control, PPE treated with vehicle and PPE treated with thymol, carvacrol or *p*-cymene groups stained with HE (E to K), with picro-sirius to collagen detection (L to P), as well as immunostained to detect MMP-9 (Q to U) and TIMP-1 (V to Z) positive cells. The PPE group showed large alveolar spaces with intense collagen deposition and increased positive cells to MMP-9 and TIMP-1 (F, M, R and W) compared to control and to treated groups. 

### 2.5. The Monoterpenes Probably Acts in Reduction of Emphysema and Lung Inflammation in Mice by Reducing Oxidative Stress and in NF-κB Expression in Lung

**Figure 5:**
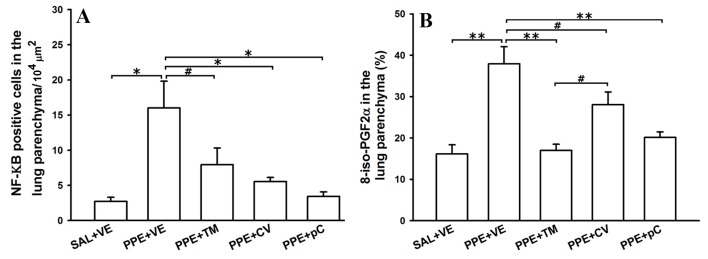

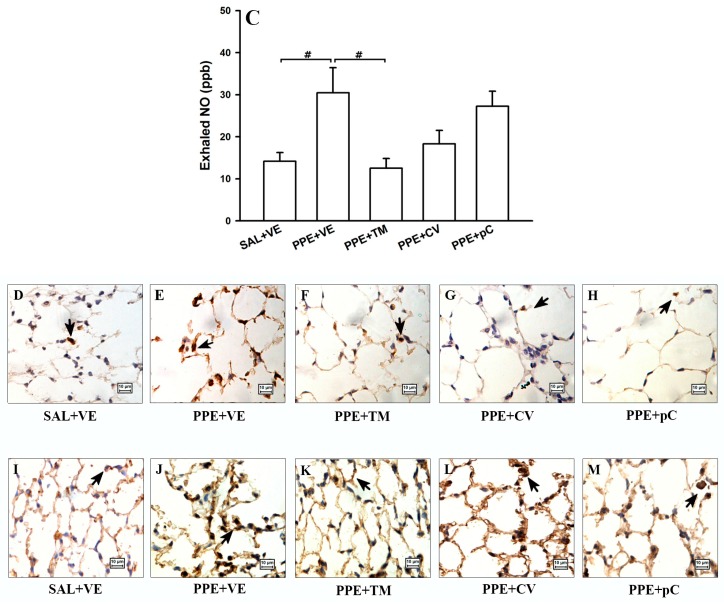
Effects of monoterpenes thymol, carvacrol and *p*-cymene in the NF-κB expression in alveolar septa and oxidative stress pathway. Data are presented as mean ± standard error (SE) (*n* = 5 to 7 mice/group) of the number of positive cells to NF-κB (**A**) detected in alveolar septa, the volume proportion of 8-iso-PGF-2α expression (**B**) detected in alveolar septa and exhaled nitric oxide detected in the exhaled air (**C**) from mice in the day 28 of the protocol. Panels **D** to **H** represent tissue stained to detect NF-κB and panels **I** to **M** to detect isoprostane by immunohistochemistry. Arrows represent positive cells in lung tissue. SAL + VE: control group with vehicle treatment; PPE + VE: porcine pancreatic elastase instillation and vehicle treatment; PPE + TM: porcine pancreatic elastase instillation and thymol treatment; PPE + CV: porcine pancreatic elastase instillation and carvacrol treatment; PPE + pC: porcine pancreatic elastase instillation and *p*-cymene treatment. * *p* < 0.01, ** *p* < 0.001 and ^#^ *p* < 0.05.

In order to understand some possible mechanisms involved in the effects of monoterpenes *p*-cymene, carvacrol and thymol in lung emphysema, the expression of the subunit p-65-NF-κB in lung parenchyma by immunohistochemistry was evaluated (Figure 5). This subunit is necessary to activate the NF-κB gene [10], which indirect reflects the activation of this pathway. The obtained results indicated that PPE induced an increase in the number of NF-κB positive cells compared with SAL + VE group (*p* < 0.01) which was attenuated by monoterpene treatment (*p* < 0.01 for carvacrol and *p*-cymene and *p* < 0.05 for thymol) (Figure 5A). Other possible mechanism involved in the action of tested monoterpenes is associated to reduction of oxidative stress, since these compounds are anti-oxidants. In this context, was evaluated the volume proportion of isoprostane expression (8-iso-PGF2α) in lung tissue. In this aspect, was observed an increase in the expression of isoprostane in PPE + VE animals compared with SAL + VE group (*p* < 0.001) (Figure 5B) which is also attenuated by monoterpenes treatments (thymol/*p*-cymene *p* < 0.01 and carvacrol *p* < 0.05). Carvacrol-treated animals also presented high levels of isoprostane compared to thymol (*p* < 0.05). The detected nitric oxide in exhaled air (ENO) is a useful marker of both pulmonary inflammation and oxidative stress. Animals that received PPE instillation and treated with vehicle (PPE + VE) showed high levels of ENO compared with SAL + VE group (*P* < 0.05) (Figure 5C). In addition, only thymol treated animals showed lower valued of ENO compared to ELA + VE (*p* < 0.05).

The photomicrographs represent the lung tissue of one control animal, PPE treated with vehicle and PPE treated with thymol, carvacrol or *p*-cymene group stained with immunohistochemistry to detect positive cells to NF-κB (D to H) and 8-isoprostane (I to M).

## 3. Discussion

PPE instillation in mice is a useful model to reproduce quickly several features of human emphysema [33,34] and PPE instillation increased macrophages as well as IL-6, KC, IL-1β and IL-17 levels in BALF. PPE also induced lung destruction, collagen deposition and an imbalance between MMP-9 and TIMP-1 expression. These changes were associated to high levels of nitric oxide detected in exhaled air and increase the isoprostane expression in lung tissue. Finally, the number of positive cells to NF-κB in lung was increased in PPE-instilled animals. Most of these features observed in this model have been previously reported in the literature [35,36] and some of these alterations have been already present 60 min after the PPE instillation [37].

In the world, 65 million people have moderate to severe COPD [38] and although there is a range of available drugs, the efficacy of many existing anti-inflammatory drugs have been disappointing in COPD patients. In this context, compounds derived from plants must be investigated as an additional potential therapeutic approach, particularly those which present anti-oxidant effects, since oxidative stress is one of the primary mechanisms involved in the development of COPD [39].

The present study shows that the monoterpenes *p*-cymene, carvacrol and thymol, extracted from essential oil from leaves of *L. sidoides*, reduced the inflammatory changes and the alveolar enlargement observed in a murine emphysema model. These changes could be associated, at least in part, with reduced NF-κB expression and cytokine release as well as the control of oxidative stress. Moreover, these treatments also attenuated the MMP-9 expression in cells present in alveolar septa. 

Increased number of inflammatory cells, such as neutrophil and macrophage, are presented in the lung and in BALF from COPD patients [2]. In animals models, the activation and increase in the number of neutrophils are observed particularly in the early phase. Other authors have found increased neutrophils only four days after elastase instillation, whereas 21 or 28, the mainly was characterized by the presence of macrophages in BALF [40,41]. The treatment with monoterpenes was able to reduce the number of macrophage recovered in the BALF in the present study. 

Macrophage is an important cell involved in emphysema physiopathology and is able to release several inflammatory mediators involved in the destruction of the alveolar parenchyma [42,43,44]. Tumor necrose factor (TNF) –α, interferon-γ and various classes of interleukins as the IL-1β, IL-6, IL-8, IL-10, IL-17, IL-18, IL-32 and others are involved in the progression of emphysema [4,45]. The monoterpenes reduced both levels of IL-6, and KC which is homologous with IL-8 in humans [46]. Increase levels of IL-6 and IL-8 were detected during COPD exacerbation in serum and in induces sputum and in BALF [47,48]. 

The IL-17, and its receptor IL17R, was first identified in CD4 + T cells. However, the IL-17, a pro-inflammatory cytokine, has become of a great interest because it is produced by a specifically cell subtype called Th17. The IL-17 pathway is involved in the host defense and plays a critical role in both acute and chronic inflammatory diseases. IL-17 is involved in either benefic or malefic effects for health protective because it can exacerbate immune responses and is produced highly by patients with chronic inflammatory diseases [49,50]. Moreover, some authors demonstrated that IL-17 is involved mainly in sustaining inflammatory responses rather than to initiate an inflammatory reaction [51]. In lung, the blockade of IL-17R reduced airway inflammation and hyperreactivity in an asthma model [52]. 

The expression of IL-17 has previously been shown in COPD patients and it seems that IL-17A induces the secretion of neutrophils-recruiting chemokines such as the expression of IL-6 in bronchial epithelial cells and fibroblasts [6,40,53] as well as increase IL-8 production [54]. More importantly, the levels of IL-17 were inversely correlated with lung function [55] in patients with COPD. Monoterpenes *p*-cymene, carvacrol and thymol reduced IL-17 levels in BALF. Kurimoto et al. [40] have shown that IL-17 is necessary to elastase-induce emphysema in experimental models. Interestingly, we also found a reduction in the levels of IL-1β induced by monoterpenes treatment. The IL1-β can be released by monocytes, macrophages and fibroblast and is increased in the peripheral blood, in sputum and in BALF from COPD patients [4]. In an experimental model, Lappalainen et al. [3] demonstrated that genetically modified mice, which expressed human IL-1β, developed an intense inflammatory process characterized by increased neutrophils and macrophages as well as emphysema.

Other authors showed the effects of other monoterpenes in the reduction of inflammation in experimental lung diseases [56,57,58,59,60]. In this regard, Boskabady and Gholami [57] showed that *Zataria multiflora* and it main constituent carvacrol reduced the inflammatory cells recovered from BALF and the levels of IL-8 in an emphysema model. Zhou et al. [60] using an asthma model showed that thymol isolated from *L. sidoides* reduced inflammatory cells in airways and the levels of Th2 cytokines in BALF. Sun et al. [58] observed that the monoterpene paeoniflorin reduced inflammation and cytokines after OVA exposure. Recently, Zhao et al. [59] showed that 1,8-cineol reduced TNF-α and IL-1β levels in BALF in an acute lung injury model. Finally, Xie et al. [56] showed that *p-*cymene reduced inflammatory cells and cytokines in ALI model. However, no data were found comparing the biological effects of structural related monoterpenes in emphysema. 

The imbalance between proteases and anti-proteases can contribute to the lung destruction in emphysema. The obtained results showed that monoterpenes thymol, carvacrol and *p*-cymene attenuate alveolar destruction in this model. Moreover, these treatments reduced collagen deposition in alveolar septa, suggesting that the reduction in lung destruction and inflammation could avoid the fibers remodeling. It is well known that MMP-9 is increased in blood, sputum and BALF from COPD patients [61,62]. Additionally, was found a reduction in positive cells to MMP-9 induced by monoterpenes treatment in PPE animals. The activity of MMP-9 is controlled by TIMP-1 [63] and although in this study the monoterpenes reduced MMP-9, this treatment did not affect TIMP-1 positive cells. These findings suggest that monoterpenes contribute to the balance between MMP-9 and TIMP-1 since it reduced the MMP-9 but maintained the levels of TIMP-1 in lung, avoiding the tissue destruction and consequently remodeling. Although other authors have shown the remodeling process in PPE models, few studies have evaluated the effects of monoterpenes in this process. 

The NF-κB is a signaling pathway that positive regulates pro-inflammatory cytokines in lung diseases [64,65]. Monoterpenes thymol, carvacrol and *p*-cymene reduced the positive cells to NF-κB in lung and it justified the effects of these compounds in reducing inflammation. In COPD, the NF-κB activation could occur in response to increase levels of IL-1β [66] and the reduction of IL-1β observed by monoterpenes treatment could justified the effects in NF-κB. 

The NF-κB pathway is regulated by redox system and could be influenced by an unbalance between oxidant and anti-oxidants enzymes [67]. The isoprostane 8-PGF-2α is a product formed in the lipid peroxidation and could be used as an indirect marker of oxidative stress, being elevated in COPD patients [68,69] and in emphysema models [18,70,71]. Interestingly, only thymol reduced the exhaled nitric oxide, which has been used as a marker to both detect the levels of lung inflammation and oxidative stress [72]. A possible explanation could be related to the contribution from constitutive isoforms in ENO, which could be modified for one compound or the other. However these finding should be detailed investigated in the future. 

The monoterpenes *p*-cymene, carvacrol and thymol are bioactive molecules with anti-inflammatory potential [73], which could be associated to anti-oxidant activity. In this case, thymol and carvacrol displayed higher potential in comparison to *p*-cymene since these are phenolic compounds and showed one hydroxyl group bearing to benzene ring, which could be associated to the capacity of established free radicals preventing the lipid peroxidation [26,27]. Interestingly in this study was observed an isoprostane reduction in lung tissue after treatment with these three related compounds suggesting that the structural differences (presence of absence of hydroxyl group in the molecular structure) do not interfere significantly in biological effects in this model. This could be partially explained by metabolization of *p*-cymene in vivo affording active carvacrol and other oxygenated derivatives [74]. Previous studies have reports the effects of carvacrol in a model of COPD and showed that the increase in thiol group in COPD animals is the main mechanism involved in the benefic effects of carvacrol [57].

It is also important to consider that the effects of thymol and carvacrol could occur in the elastase activity, an enzyme that displayed an important role in the development of emphysema [75,76,77]. Even considering that the effects of these compounds are in the elastase activity, the clinical interest is relevant since the direct action of a drug in the activity of elastase could be benefic to COPD patients since this enzyme is involved in the progression and development of the disease. 

**Figure 6:**
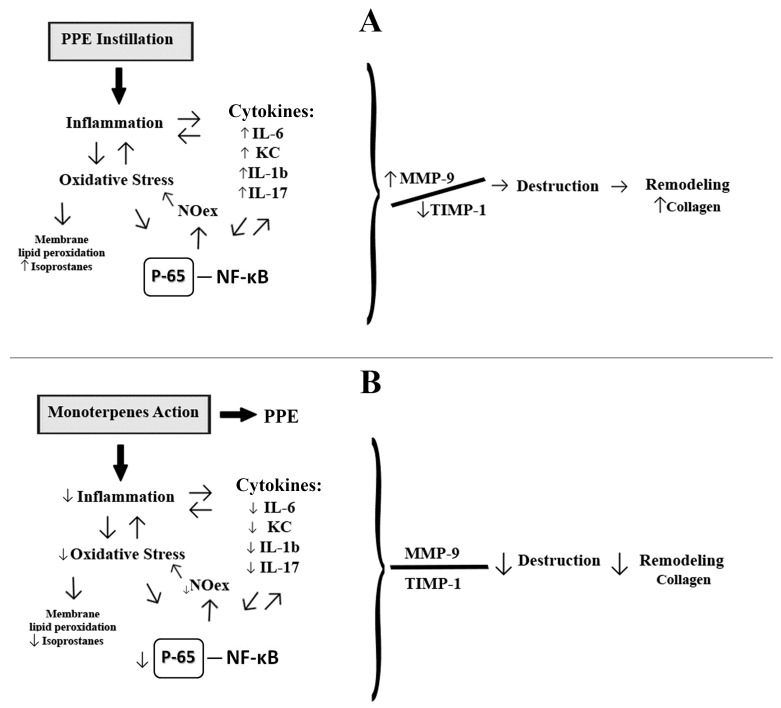
Monoterpenes probably mechanism is related to reduction in inflammation, oxidative stress and in NF-κB in lung of PPE-instilated animals. (**A**) The PPE instillation induces inflammation and increase marker of oxidative stress. Cytokines such as IL-6, KC, IL-1β and IL-17 are released and induce the perpetuation of inflammation. Together with oxidative stress, there is activation in NF-κB pathway, which in turn up-regulated transcription of proinflammatory cytokines and iNOS, that can induce an increase in exhaled nitric oxide. Augment in MMP-9 positive cells can amplify inflammation and contribute to the destruction of lung tissue culminating with collagen fibers deposition; (**B**) The administration of monoterpenes led to a reduction of the inflammatory process and consequent reduction in oxidative stress, which is related to the effects of monoterpenes in cytokines reduction (IL-6, KC, IL-1b and IL-17), NF-κB and isoprostane. These anti-inflammatory and antioxidant effects contributed to reduction in tissue destruction.

In conclusion, the results of the present study indicate a preventive effect of monoterpenes on lung emphysema by controlling the inflammation, avoiding the lung remodeling through NF-κB and isoprostane reduction (Figure 6). These findings could be of great clinical interest suggesting a potential role of monoterpenes on lung inflammation and oxidative stress in COPD.

## 4. Materials and Methods

### 4.1. General Experimental Procedures

^13^C-NMR spectra were recorded 75 MHz in an Ultrashield 300 Advance III spectrometer (Bruker, Fremont, CA, USA) using CDCl_3_ (Tedia Brazil, Rio de Janeiro, Brazil) as solvent and TMS (Aldrich, St. Louis, MO, USA) as internal standard. Silica gel for flash chromatography (Merck, Kenilworth, NJ, USA, 230–400 mesh) and silica gel 60 F_254_ (Merck, Kenilworth, NJ, USA) were used for column chromatography and for analytical (0.25 mm) TLC, respectively. GC-FID chromatograms were obtained on a GC-2010 gas chromatograph (Shimadzu, Kyoto, Japan) equipped with an flame ionization detector (FID) and one automatic injector AOC-20i (Shimadzu, Kyoto, Japan) using RtX-5 capillary column (5% phenyl, 95% polydimethylsiloxane (Restek, Bellefonte, PA, USA, 30 m × 0.32 mm × 0.25 μm film thickness). These analyses were performed by injecting 1.0 μL of a 1.0 mg/mL solution of sample material in pentane in a split mode (1:10) employing helium as the carrier gas (1 mL/min) under the following conditions: injector and detector temperatures of 250 °C and 280 °C, respectively; oven programmed temperature from 100–260 °C at 5 °C/min, holding 15 min at 260 °C. LREIMS analysis was carried out using a MS-QP-5050A mass spectrometer (Shimadzu, Kyoto, Japan), operating under an ionization voltage of 70 eV and an ion source temperature of 230 °C. 

### 4.2. Chemical and Reagents

All solvents used were of analytical grade and purchased from CAAL (São Paulo, Brazil) while elastase was obtained from Sigma (St. Louis, MO, USA, 6.6 units/mg, E1250, type I). ELISA and antibodies were purchased from R&D Systems (Minneapolis, MN, USA).

### 4.3. Plant Material

*L. sidoides* was collected in the Atlantic Forest Biome in São Paulo State, Brazil, in March, 2014. The studied species was identified by Prof. Euder G.A. Martins and its identification of was possible comparing the voucher specimens with those deposited in the Herbarium of the Universidade de São Paulo—SP.

### 4.4. Essential Oil Extraction

Fresh leaves (200 g) were subjected to hydrodistillation in a Clevenger type apparatus for 4 h. After extraction using CH_2_Cl_2_ the essential oil was dried using anhydrous Na_2_SO_4_, filtered and the solvent was evaporated under reduced pressure to afford 644 mg of crude essential oil (yield 0.32%). 

### 4.5. Isolation of Monoterpenes

Part of the crude essential oil of *L. sidoides* (400 mg) was subjected to separation over flash silica gel column chromatography eluted with CH_2_Cl_2_ and CH_2_Cl_2_:MeOH (99:1, 98:2 and 95:5). Using this procedure, were obtained 45 fractions (5 mL each) which were analyzed by GC-FID and then pooled into eight groups (**A**–**H**). Group **B** (12 mg) was composed by pure *p*-cymene (98.5%) whereas groups **D** (16 mg) and **G** (66 mg) were composed by carvacrol (purity 99.0%) and thymol (purity 99.6%), respectively. 

#### 4.5.1. *p*-Cymene

Colorless oil. ^13^C-NMR (75 MHz, CDCl_3_) δ_C_: 135.1 (C-1), 126.3 (C-2/C-6), 129.0 (C-3/C-5), 33.8 (C-7), 24.1 (C-8/C-9), 20.9 (C-10). LREIMS *m*/*z* (rel. int.): 134 (25), 119 (100), 103, 91 (28), 77, 65 (11).

#### 4.5.2. Carvacrol

Amorphous solid. ^13^C-NMR (75 MHz, CDCl_3_) δ_C_: 130.8 (C-1), 153.6 (C-2), 121.0 (C-3), 148.4 (C-4), 118.7 (C-5), 112.9 (C-6), 33.6 (C-7), 23.9 (C-8/C-9), 15.3 (C-10). LREIMS *m*/*z* (rel. int.): 150 (28), 135 (100), 115, 107 (16), 91 (20), 77 (14).

#### 4.5.3. Thymol

Amorphous solid. ^13^C-NMR (75 MHz, CDCl_3_) δ_C_: 131.3 (C-1), 126.2 (C-2), 152.5 (C-3), 136.5 (C-4), 121.6 (C-5), 116.0 (C-6), 26.6 (C-7), 22.6 (C-8/C-9), 20.8 (C-10). LREIMS *m*/*z* (rel. int.): 150 (27), 135 (100), 115 (18), 107 (15), 91 (24), 77 (12).

### 4.6. Animals

Male C57BL/6 (6–8 weeks) mice received humane care according to the “Guide for the Care and Use of Laboratory Animals” (Institute of Laboratory Animal Resources-1996, Washington, DC, USA). All surgical procedures were performed while the animals were under general anesthesia. The male mice used in the present study were maintained in a temperature-controlled room at 21–23 °C with a 12-h light/dark cycle and ad libitum access to water and food. This study was approved by the Ethics Committees of Federal University of São Paulo (São Paulo, Brazil, protocol number 715701/15) and of University of São Paulo (protocol number 029/15).

### 4.7. Experimental Design

Forty male C57BL6J mice were aleatory distributed among five experimental groups: A, SAL + VE group: animals that received nasal instillation of saline + vehicle treatment (50% DMSO + 50% Saline); B, PPE + VE group: animals that received nasal instillation of porcine pancreatic elastase (PPE) + vehicle (50% DMSO + 50% Saline) treatment; C, PPE + TM group: animals that received nasal instillation of elastase + thymol treatment; D, PPE + CV group: animals that received nasal instillation of elastase plus carvacrol treatment; and E, PPE + pC group: animals that received nasal instillation of elastase and *p*-cymene treatment. DMSO was used to dissolve the tested monoterpenes. 

### 4.8. PPE-Induced Emphysema in Mice

Mice were anesthetized with xylazine (40 mg/kg) and ketamin (5 mg/kg) and intranasal instilled with 50 μL of PPE (6.6 units/mg, E1250, type I, Sigma-Aldrich) [78] with a concentration of de 0.677 UI (day zero). Control group received an intranasal instillation of the same volume of saline. All animals were evaluated 28 days after the intranasal instillation.

### 4.9. Monoterpenes Treatment

Ten microliters of *p*-cymene, carvacrol, thymol or vehicle was administered by intranasal instillation 30 min after PPE or saline instillation, and then repeated on 7th, 14th, 21st, and 28th days of the protocol [18]. The dose of *p*-cymene, carvacrol, thymol used were 20 mg/kg chosen based in previous studies of our lab in acute lung injury models (unpublished results) diluted in DMSO and saline in 1:1 proportion. Previous studies determined that this vehicle was not toxic for animals [18,79]. 

### 4.10. Exhaled Nitric Oxide and BALF Collection

Animals were anesthetized with injection of thiopental (70 mg/kg), tracheostomized and connected to a ventilator for small animals (Harvard 683; Harvard Apparatus, South Natick, MA, USA) to collect the exhaled nitric oxide (ENO). The concentrations of ENO were measured by chemiluminescence using a fast-responding analyzer (NOA 280; Sievers Instruments, Boulder, CO, USA), as previously described [19]. We measured ENO levels at the expiratory port of the ventilator using a Mylar bag during 10 min. A NO filter was attached to the breathing circuit in order to avoid environment contamination. After NO collection, animals were exsanguinated, and the BALF was collected by washing the lungs with 0.5 mL of sterile saline and withdrawing the fluid (3 times). For total and differential cell counting, the BALF was centrifuged (Model GS-6R Centrifuge, Beckman Instruments, Fullerton, CA, USA) at 800 *g* for 8 min at 5 °C, and the cell pellet was resuspended in 0.2 mL of sterile saline. The total viable cell count was determined using a Neubauer haemocytometer chamber (Carl Roth, Karlsruhe, Germany), and differential cells (at least 300 cells) were counted using an optical microscope (Model BX40 Olympus Optical Co, Tokyo, Japan) after the cytocentrifuge preparations of the BALF (450 rpm for 6 min) (model: Cytospin 3, Shandon Instruments, Sewickley, PA, USA) and Diff-Quick staining (Biochemical Sciences Inc., Swedesboro, NJ, USA) [18]. 

The protein concentrations of IL-6, IL-8, IL-1β and IL-17 in the BALF were assayed by ELISA according to the manufacturer’s protocol for a Duo-Set kit from R&D Research and Development (San Diego, CA, USA) [18] and were expressed in pg of cytokine/mL BALF. 

### 4.11. Lung Histopathology and Immunohistochemistry

Lungs were removed in bloc, the right lung was fixed, and histological sections (3 µm thickness) were stained with hematoxylin and eosin (H&E) to evaluate the mean linear intercepts, an index that is widely used to characterize the presence of emphysema [21] or picro-sirius (Direct Red 80, CI 35780; Sigma-Aldrich) to identify collagen fibers [18]. With a 50-line, 100-point grid connected to the ocular of the microscope (Model BX40 Olympus Co.), was assessed the Mean Linear Intercepts (Lm) and the volume proportion of collagen fibers in the alveolar tissue using a point-counting technique. The mean linear intercept (Lm) was determined at a magnification of 200× in 20 non-overlapping fields of the distal lung parenchyma per animal, and the number of times that the lines of the integrating eyepiece intersected with the alveolar septum was counted. The volume proportion of collagen was determined in 10 lung fields of each animal by dividing the number of points corresponding to collagen by the total number of points corresponding to alveolar septa (400×). 

Immunohistochemistry was performed using anti-MMP-9 (goat polyclonal IgG diluted to 1:500), anti-TIMP-1 (rabbit polyclonal IgG diluted to 1:100), anti-NF-κB p65 (rabbit polyclonal igG diluted to 1:300) antibodies (Santa Cruz Biotechnology, Santa Cruz, CA, USA) and the biotin–streptavidin–peroxidase method. We used the Vectastain ABC Kit (Vector Elite PK-6105, Burlingame, CA, USA) and DAB (Sigma-Aldrich) to reveal the staining. We determined the number of cells that were positive for MMP-9, TIMP-1 and NF-κB using the point-counting technique as described above. The number of points corresponding to alveolar septa and the number of positive cells within the alveolar septa were counted, and the results were expressed as cells per unit area (10^4^ µm^2^) [18].

### 4.12. Statistical Analyses

The results were statistically analyzed using the SigmaStat (Jandel Scientific, San Rafael, CA, USA) and analysis of variance ANOVA factor was used. The data were obtained and therefore parametric test was used for post hoc Student-Newman-Keuls test. A *p* < 0.05 was considered statistically significant.
